# Intraoperative Phrenic Nerve Monitoring in Lung Transplants: Results From a Single Center Prospective Cohort

**DOI:** 10.3389/ti.2026.15400

**Published:** 2026-02-27

**Authors:** Alberto Pérez de Vargas, Lucas Rafael Pérez-Cuesta, Andrés Varela de Ugarte, Edwin Ebrat Mancilla, Alejandra Sánchez Aparicio, Marta Vaquero Martínez, Luis López Pájaro, Paula Péndola Calero, Virginia Russu, David Gómez de Antonio

**Affiliations:** 1 Clinical Neurophysiology Department Hospital Universitario Puerta de Hierro de Majadahonda, Madrid, Spain; 2 Thoracic Surgeon from Thoracic Surgery Department Hospital Universitario Puerta de Hierro de Majadahonda, Madrid, Spain

**Keywords:** lung transplant, mechanical ventilation weaning, phrenic intraoperative monitoring, postoperative nerve injury, postoperative phrenic damage

Dear editors,

Lung transplantation is a complex surgical procedure associated with significant perioperative morbidity. Adequate respiratory function after transplantation depends largely on proper diaphragmatic movement, which is exclusively innervated by the phrenic nerve. Injury to this nerve during surgical dissection, mediastinal manipulation, or thermal exposure may result in diaphragmatic paralysis or dysfunction, thereby compromising postoperative respiratory mechanics.

Phrenic nerve injury following lung transplantation has been reported with variable incidence and may significantly delay weaning from mechanical ventilation. Prolonged ventilatory support increases the risk of infection, ICU-related complications, and overall hospital costs. Early detection of phrenic nerve compromise during surgery may allow immediate corrective maneuvers and reduce the severity or permanence of nerve injury.

Intraoperative neurophysiological monitoring provides continuous functional assessment of neural pathways and allows surgeons and anesthesiologists to detect early warning signs of nerve injury [1, 2]. While its benefit has been well established in other surgical fields, experience with phrenic nerve monitoring in lung transplantation remains scarce [3]. The aim of this study was therefore to describe our institutional experience with intraoperative phrenic nerve monitoring and to evaluate its impact on postoperative outcomes and healthcare costs.

This longitudinal, prospective, parallel-group study was conducted at Hospital Universitario Puerta de Hierro Majadahonda in accordance with international ethical guidelines and the ethics statement of the International Society for Heart and Lung Transplantation (ISHLT). All patients undergoing lung transplantation between 11 January 2018, and 31 December 2019, were screened for inclusion.

Patients were excluded if they died within 4 weeks following transplantation, underwent redo lung transplantation, required preoperative non-invasive mechanical ventilation or extracorporeal membrane oxygenation (ECMO), or underwent combined heart–lung transplantation. Redo transplantations were excluded to maintain sample homogeneity, as these cases are typically more complex and associated with a higher risk of phrenic nerve injury.

A total of 58 patients met the inclusion criteria. Thirty-one patients underwent lung transplantation with intraoperative phrenic nerve monitoring (IOM group), while 27 patients formed the control group. Monitoring was applied consecutively depending on the availability of the neurophysiology team. Randomization and blinding were not performed due to ethical concerns regarding the potential withholding of a protective monitoring technique.

All patients underwent a standardized preoperative neurophysiological assessment to document baseline phrenic nerve function. Patients in the monitored group also underwent postoperative neurophysiological evaluations at 1, 6, and 12 months after transplantation.

Intraoperative phrenic nerve monitoring was performed using a combination of compound muscle action potential (CMAP) recordings and free-running electromyography (EMG). CMAPs were obtained by electrical stimulation of the phrenic nerve in the cervical region, with recordings obtained from the diaphragm using both external and internal electrodes [4].

Baseline recordings were established at the beginning of the surgical procedure and served as a reference throughout the operation. Alarm criteria were defined as a permanent reduction in CMAP amplitude greater than 50% or an increase in distal latency greater than 10% relative to baseline values [5]. Lesser changes, such as amplitude reductions between 30% and 50%, were considered warning signs and prompted immediate communication with the surgical team [6].

Free-running EMG was used to detect mechanical or thermal irritation of the nerve in real time. In addition, train-of-four (TOF) stimulation was employed to monitor the degree of neuromuscular blockade and exclude anesthetic interference with CMAP amplitude. Throughout the procedures, neuromuscular blockade was carefully adjusted to maintain reliable and reproducible recordings.

No adverse events related to intraoperative monitoring were observed. Baseline demographic and clinical characteristics were comparable between the two groups, with the exception of a higher prevalence of diabetes mellitus in the monitored group. Importantly, none of the patients with diabetes demonstrated evidence of preoperative peripheral neuropathy on baseline testing.

Postoperative outcomes demonstrated a consistent trend toward improved recovery in the monitored group. Patients who underwent intraoperative monitoring experienced shorter durations of mechanical ventilation, reduced ICU stay, and shorter overall hospital stay compared with controls. Although these differences did not reach statistical significance, they were clinically meaningful (Table 1).

**TABLE 1:** Post-surgery variables comparative.

Variables	Group 1 (IOM)	Group 0 (control)	p
N	31	27	​
ICU_st	11.35 ± 12.05	14.40 ± 15.1	0.39
MVT	6.77 ± 13.68	11.92 ± 20.01	0.13
Early extubation	14 (45.16%)	8 (29.62%)	0.11
Hospital_st	46.74 ± 19.69	69.48 ± 60.70	0.05
Total cost (euros)	37.226,91	51.179,76	0,09

The quantitative variables are expressed as mean standard ± deviation. The qualitative variables are expressed as absolute variables and percentages. IOM: intraoperative monitorization, ICU_st: stay in ICU, SD: standard deviation, MVT: mechanical ventilation time, n: number of cases, %: percentage, Hospital_st: hospital stay. Total cost per patient.

Seven cases of new-onset phrenic nerve dysfunction were detected postoperatively, most of which were transient and associated with reversible intraoperative changes. One patient exhibited a sustained reduction in CMAP amplitude greater than 50%, which correlated with persistent phrenic nerve dysfunction at 1-year follow-up.

Cost analysis revealed an average saving of €13,952.84 per patient in the monitored group, primarily attributable to reduced ICU and hospital length of stay.

The present study demonstrates that intraoperative phrenic nerve monitoring during lung transplantation is feasible, safe, and capable of providing clinically relevant information. Early detection of intraoperative changes allowed the surgical team to implement corrective maneuvers aimed at preventing permanent nerve injury [7].

When warning criteria were met, recovery strategies summarized by the acronym TIPP (Time, Irrigation, Papaverine, Pressure) were applied [8]. The most frequently used interventions were temporary interruption of the surgical maneuver and irrigation with warm saline, both of which were effective in restoring baseline CMAP values in the majority of cases.

Our findings support previous observations suggesting that CMAP amplitude reductions greater than 50% are associated with severe and potentially permanent nerve injury, whereas reductions between 30% and 50% may result in mild to moderate dysfunction. Even mild phrenic nerve dysfunction was associated with prolonged hospital stay, underscoring the clinical relevance of early detection [9].

Although the study was not powered to demonstrate statistically significant differences in all clinical endpoints, the consistent trend toward improved outcomes and the substantial cost savings observed highlight the potential value of routine intraoperative monitoring.

Intraoperative phrenic nerve monitoring during lung transplantation represents a valuable adjunct to surgical care. The technique is safe, provides real-time functional information, and may reduce postoperative morbidity, length of hospital stay, and overall healthcare costs. Based on these findings, routine implementation of phrenic nerve monitoring should be considered in lung transplantation programs [10].

## Data Availability

The raw data supporting the conclusions of this article will be made available by the authors, without undue reservation.
